# A Population-Based Cohort Study of All-Cause and Site-Specific Cancer Incidence Among Patients With Type 1 Diabetes Mellitus in Taiwan

**DOI:** 10.2188/jea.JE20140197

**Published:** 2015-09-05

**Authors:** Pei-Chun Hsu, Wei-Hung Lin, Te-Hui Kuo, Hui-Mei Lee, Chieh Kuo, Chung-Yi Li

**Affiliations:** 1 Department and Graduate Institute of Public Health, College of Medicine, National Cheng Kung University, Tainan, Taiwan; 2 Department of Internal Medicine, National Cheng Kung University Hospital, College of Medicine, National Cheng Kung University, Tainan, Taiwan; 3 Institute of Clinical Medicine, College of Medicine, National Cheng Kung University, Tainan, Taiwan; 4 Department and Graduate Institute of Nursing, College of Medicine, Kaohsiung Medical University, Kaohsiung, Taiwan; 5 Division of Cardiology, Department of Medicine, Sin-Lau Hospital, Tainan, Taiwan; 6 Department of Public Health, College of Public Health, China Medical University, Taichung, Taiwan

**Keywords:** type 1 diabetes mellitus, neoplasm, cohort studies, incidence densities, standardized incidence ratio

## Abstract

**Background:**

The relationship between type 1 diabetes mellitus (T1DM) and cancer incidence remains unclear. We sought to assess the all-cause and site-specific cancer incidence in patients with T1DM.

**Methods:**

A retrospective cohort study design was employed, in which 14 619 patients with T1DM were retrieved from Taiwan’s National Health Insurance medical claims between 2000 and 2007. The study subjects were followed to the end of 2008, and cancer incidence was assessed. We calculated age-, sex-, and calendar year-standardized incidence ratios (SIRs) of all-cause cancer incidence and site-specific neoplasm incidence, with reference to the general population.

**Results:**

Seven hundred and sixty patients were identified for all-cause cancer over 86 610 person-years, representing an incidence rate of 87.75 cases per 10 000 person-years. The incidence rate was higher in males than in female patients (109.86 vs 69.75 cases per 10 000 person-years). T1DM was associated with a significantly increased SIR of all-cause cancer (1.13; 95% confidence interval [CI], 1.05–1.22). The sex-specific SIR was significantly elevated in female patients (1.19; 95% CI, 1.07–1.33), but the SIR for male patients was insignificantly elevated (1.09; 95% CI, 0.99–1.20). Pancreatic cancer showed the greatest increase in SIR among both male and female patients with T1DM. Male patients experienced significantly increased SIRs for kidney, rectum, liver, and colon neoplasm, and significantly increased SIRs were noted for ovarian, bladder, and colon cancer in female patients.

**Conclusions:**

T1DM was associated with a 13% increase in risk of all-cause cancer incidence. Patients with T1DM should be advised to undergo cancer screening for certain types of cancer.

## INTRODUCTION

There is increasing epidemiological evidence demonstrating positive associations between diabetes mellitus (primarily type 2 diabetes mellitus [T2DM]) and certain site-specific cancers, such as those of the liver, pancreas, endometrium, colon/rectum, breast, and bladder.^1^ The observed link between diabetes and cancer could be due to potential risk factors common to both diabetes and cancer, including age, sex, obesity, physical inactivity, diet, alcohol consumption, and smoking.^1^^–^^3^ Additionally, diabetes may also lead to hyperinsulinemia,^4^ hyperglycemia,^5^^,^^6^ or chronic inflammation,^7^^,^^8^ which are all considered to be biological mechanisms by which a neoplastic process may occur in patients with diabetes.

While the majority of the research to date has focused on the putative link between T2DM and cancer incidence, very few studies have explored cancer incidence in patients with type 1 diabetes mellitus (T1DM). The validity of extrapolating excess cancer risk noted in patients with T2DM to those with T1DM is questionable, mainly due to the fact that patients with T1DM are often younger and less obese than those with T2DM. A recent nationwide study conducted in Taiwan estimated that the annual number of patients with T1DM increased from 3939 in 2000 to 8043 in 2009. The ratio of T1DM to all diabetes ranged from 0.56% to 0.66% during the same period.^9^ An earlier national mass screening showed that the ratio of newly diagnosed type 2 to type 1 diabetes among school children aged 6–18 years is approximately six to one.^10^ The proportion of patients with insulin resistance was also low among T1DM patients.^11^ A recent review^3^ summarized findings from four cohort studies^12^^–^^15^ and two case-control studies^16^^,^^17^ that investigated the cancer incidence in T1DM patients and concluded that current epidemiological research investigating the link between T1DM and cancer has resulted in mixed findings, mainly due to dissimilarities in the research methods used. The methodological concerns included criteria used to determining T1DM, the potential latency most relevant to the cancer incidence in relation to T1DM, and limited sample sizes of both T1DM and cancer patients in site-specific analyses.^3^

A recent Taiwanese study of 7225 incident cases of T1DM from 1999 to 2010 showed that the age- and sex-standardized bi-annual incidence rate of T1DM rose from 1999–2000 (2.63 cases per 100 000) to 2003–2004 (3.22 cases per 100 000) and then rose mildly thereafter to 2009–2010 (3.34 cases per 100 000).^18^ In addition, cancer has been the leading cause of death in Taiwan since 1982, and the cancer incidence rate is still growing.^19^ Given the rising incidence of both diabetes and cancer and inconsistencies in previous study findings, we undertook this cohort study to further explore the nature of the relationship between T1DM and cancer.

## METHODS

### Source of datasets

Data analyzed in this study were retrieved from the medical claims of the National Health Insurance Research Database (NHIRD) provided by Taiwan’s Ministry of Health and Welfare National Health Insurance Administration (NHIA). The NHIRD provides all inpatient and ambulatory medical claims for around 99% of Taiwanese people.^20^ To ensure the accuracy of claim files, the Bureau of National Health Insurance performs quarterly expert reviews on a random sample for every 50 to 100 ambulatory and inpatient claims.^21^ Therefore, information obtained from the NHIRD is considered to be relatively complete and accurate.^22^^,^^23^ In this study, we retrieved ambulatory care visit claims (ACVCs), inpatient claims (ICs), and registry for beneficiaries (RB) from the NHIRD. Access to NHIRD has been reviewed and approved by the Review Committee of the National Health Research Institutes. The study has also obtained the institutional review board approval from the National Cheng Kung University Governance Framework for Human Research Ethics (#102-127).

### Study design and identification of study subjects

This is a retrospective cohort study in which the potential study cohort consisted of all patients who sought ambulatory care visit for T1DM (International Classification of Disease 9th version, Clinical Modification [ICD-9-CM] codes 250x1 or 250x3) between 2000 and 2007. In Taiwan, NHIA issues catastrophic illness/injury certificates (CIC) to all patients who suffer from T1DM, and these patients are exempt from copayment to the National Health Insurance (NHI) plan if they seek care for T1DM. To issue the CIC for T1DM, the diagnosis must be recertified by specialists according to clinical manifestation, biochemistry, and the presence of auto-antibodies. A recent validation study demonstrated a positive predictive value of 98.3% for the T1DM diagnosis with CIC that appears in the NHIRD.^18^

In order to obtain valid estimates of cancer incidence, we excluded patients with discharge codes of cancer (ICD-9-CM codes 140–208) between 1997 and 1999. We finally included 14 619 T1DM patients who sought diabetes care with CICs in 2000–2007. Among the study cohort, 9745 (66.7%) patients were diagnosed before 2000 (ie, prevalent cases). The number of annual incident T1DM cases in 2000 to 2007 was 590, 602, 609, 713, 645, 591, 568, and 556, respectively. Because of unavailability of medical claims for 1996 and earlier years, the information on the exact duration of T1DM for all study subjects were not obtainable.

### Follow-up and end-points

Using the unique personal identification numbers, we linked study subjects to the IC from 2000 to 2008, identifying, if any, the first episode of malignant neoplasm (ICD-9-CM codes 140–208) as the end points of this study. Cancer is one of 31 categories of CICs issued by the NHIA. To approve a cancer diagnosis with CIC, the attending physician should provide pathology evidence and imaging studies, including radiographs, bone scans, and CT or MRI scans, supporting the diagnosis of cancer. The records are further reviewed by a committee, and only patients who meet the criteria for the diagnoses are issued a CIC.^24^ The index date for each study subject of T1DM was the date of his/her first appearance at medical claim for T1DM between 2000 and 2007. All study subjects were followed from the index date to the occurrence of an end point (ie, cancer ambulatory care or admission), withdrawal from the insurance system (almost always due to death), or December 31, 2008, whichever came first.

### Statistical analysis

We first calculated the age- and gender-specific rates of all-cause cancer incidence in study subjects estimated with person-years as the unit of measurement. Second, we compared the cancer incidence rate of patients with T1DM with that of the general population in Taiwan. To perform such comparisons, the expected cancer incidence in T1DM patients was calculated via the person-year approach, using the calendar year (2000–2008), age groups (<30, 30–44, 45–59, and ≥60 years), and sex-specific annual rates of all-cause or site-specific cancer ambulatory care/hospitalization with reference to the general population. Information regarding the cancer ambulatory care/hospitalization of the general population was also retrieved from the NHI program between 2000 and 2008. Age-, sex-, and calendar year-standardized incidence rates (SIRs) were calculated by dividing the observed number of cancer hospitalizations by the expected number.

The cancer sites selected in the current analysis were mainly those previously reported to be associated with T2DM, including colorectal, pancreatic, breast, liver, prostate, stomach, lung, bladder, and kidney.^25^^,^^26^ Certain types of cancer that are relatively common in women, such as ovary, endometrial, and cervical neoplasm, were also included in the analysis. The 95% confidence intervals (CIs) for SIRs were estimated using the Byar approximation proposed by Breslow and Day.^27^ The analyses were performed using EGRETs (Cytel Software Corporation, Cambridge, MA, USA), and significance was accepted at *P*-value of less than 0.05.

## RESULTS

Female patients slightly dominated the study cohort (7752 women and 6867 men). Male patients were slightly older than female patients (51.3 [standard deviation {SD} 21.2 years] vs 49.2 [22.1] years). The proportions of male patients with T1DM aged <30, 30–44, 45–59 and ≥60 years were 18.0%, 14.8%, 26.0%, and 41.0%, respectively, whereas the corresponding figures for female patients were 24.6%, 14.60%, 21.4%, and 39.5%. The mean (SD) duration of follow-up was 5.67 (3.40) for male patients and 6.16 (3.16) years for female patients. Seven hundred and sixty patients were hospitalized for all-cause cancer over 86 610 person-years, representing an incidence rate of 87.75 cases per 10 000 person-years (95% CI, 87.13–88.37 cases per 10 000 person-years). The incidence rate of all-cause cancer was estimated at 109.86 (95% CI, 108.81–110.90) and 69.75 (95% CI, 69.00–70.50) cases per 10 000 person-years for male and female patients with T1DM, respectively (Table 1).

**Table 1.** Sex- and age-specific incidence rates and 95% confidence intervals of all cancer in patients with type 1 diabetes in Taiwan, 2000–2008

Sex and age	Number of incident cancers	Person-years observed	Incidence rate	

			Estimate^a^	95% CI
Overall (years)				
<30	11	17 047	6.45	6.07–6.83
30–44	39	12 071	32.31	31.29–33.32
45–59	194	18 420	105.30	103.84–106.80
≥60	516	39 074	132.06	130.92–133.20
All	760	86 610	87.75	87.13–88.37
Male (years)				
<30	6	6309	9.51	8.75–10.51
30–44	23	4984	46.15	44.26–48.03
45–59	100	9554	104.67	102.62–106.72
≥60	298	18 023	165.34	163.47–167.22
All	427	38 869	109.86	108.81–110.90
Female (years)				
<30	5	10 738	4.66	4.25–5.06
30–44	16	7087	22.58	21.47–23.68
45–59	94	8866	106.02	103.88–108.17
≥60	218	21 051	103.56	102.18–104.93
All	333	47 741	69.75	69.00–70.50

CI, confidence interval.

^a^Per 10^4^ person-years.

Compared to the general population, patients with T1DM had a significantly increased rate of all-cause cancer incidence, with an SIR of 1.13 (observed vs expected number of incident cancer cases: 760.0 vs 671.1) and 95% CI of 1.05–1.22 (not shown in table). A significantly elevated age- and calendar year-SIR of all-cause cancer incidence was noted for female patients (1.19; 95% CI, 1.07–1.33), but the elevated SIR of all-cause cancer incidence was only marginally significant for male patients (1.09; 95% CI, 0.99–1.20) (Table 2).

**Table 2.** Overall and site-specific standardized incidence ratio of cancer in patients with type 1 diabetes in Taiwan, 2000–2008

Cancer site (ICD-9-CM codes)	Male				Female			
	
	Number of incident cancers		Standardized incidence ratio^a^		Number of incident cancers		Standardized incidence ratio	
			
	Observed	Expected	Estimate	95% CI	Observed	Expected	Estimate	95% CI
Stomach (151)	17	15.8	1.08	0.63–1.72	14	10.5	1.33	0.73–2.24
Colon (153)	31	20.6	1.50	1.02–2.14	39	20	1.95	1.39–2.67
Rectum (154)	27	16.5	1.64	1.08–2.38	15	14	1.07	0.60–1.77
Liver (155)	92	56.6	1.63	1.31–1.99	30	29.9	1.00	0.68–1.43
Pancreas (157)	12	4.6	2.61	1.35–4.56	16	3.9	4.10	2.35–6.66
Lung (162)	51	38.7	1.32	0.98–1.73	35	25.3	1.38	0.96–1.92
Prostate (185)	66	21.2	3.11	2.41–3.96				
Bladder (188)	17	37.8	0.45	0.26–0.72	11	3.8	2.89	1.45–5.18
Kidney (184, 189)	11	5.2	2.12	1.06–3.79	13	8.6	1.51	0.80–2.58
Female breast (174)			NA		56	54.1	1.04	0.78–1.34
Cervix uteri (180)			NA		27	19.2	1.41	0.93–2.05
Endometrial (179, 182)			NA		12	6.32	1.90	0.98–3.32
Ovary (188)			NA		7	1.8	3.89	1.56–8.01

All-cause	427	392.0	1.09	0.99–1.20	333	279.1	1.19	1.07–1.33

CI, confidence interval; NA, not applicable.

^a^Standardized for age and calendar year.

Table 2 also shows age- and calendar year-SIRs of site-specific cancer incidence for both male and female patients with T1DM. Male patients had the highest site-specific SIR for prostate neoplasm (3.11; 95% CI, 2.41–3.96). Male patients also had significantly increased SIRs for cancer of the pancreas (2.61; 95% CI, 1.35–4.56), kidney (2.12; 95% CI, 1.06–3.79), rectum (1.64; 95% CI, 1.08–2.38), liver (1.63; 95% CI, 1.31–1.99), and colon (1.50; 95% CI, 1.02–2.14), but had significantly reduced SIR for bladder cancer (0.45; 95% CI, 0.26–0.72). For female patients, significantly increased SIRs were noted for cancer of the pancreas (4.10; 95% CI, 2.35–6.66), ovary (3.89; 95% CI, 1.56–8.01), bladder (2.89; 95% CI, 1.45–5.18), and colon (1.95; 95% CI, 1.39–2.67).

## DISCUSSION

Information from this study may advance our understanding regarding the effect of T1DM on cancer incidence in Taiwan, as potential geographic variations in diagnosis and treatment of both T1DM and cancer might modify the relationship between T1DM and cancer.^3^ Our study reported a 13% increase in risk of all-cause cancer in T1DM, which was similar to the elevated risk (10%–20%) observed in one Danish study^14^ and two Swedish studies^12^^,^^15^ but lower than that reported in an earlier Danish study.^13^ The gender-specific analysis showed significantly increased SIR (1.19; 95% CI, 1.07–1.33) for female patients, but the SIR (1.09; 95% CI, 0.99–1.20) for male patients was only slightly, but insignificantly, elevated. The highest site-specific SIR was for prostate cancer in men (SIR 3.11; 95% CI, 2.41–3.96) and pancreas cancer for women (SIR 4.10; 95% CI, 2.35–6.66). However, interpretations of the study findings should be cautious because the study had a short period of follow-up, which may have yielded unreliable risk estimates. The findings on site-specific cancer risk also need to be interpreted cautiously because of the very small number of incident cancers in the majority of cancer sites. The risk estimates would be more robust after an extended period of follow-up in the future.

The sex differences in the relationship between T1DM and cancer varied in previous reports. In our study, however, we found that female patients with T1DM experienced a greater SIR than males of all-cause cancer incidence. Such results were inconsistent with the findings from one earlier Swedish study^13^ but were similar to those of two more recent studies.^12^^,^^28^ Green and Jensen followed 1499 insulin-treated individuals and compared their cancer incidence with the general population. The results showed that the relative risks (RRs) of all-cause cancer for men and women were 1.47 (95% CI, 1.03–1.83) and 1.08 (95% CI, 0.77–1.51). Detailed stratified analyses further showed that only men <55 years of age had a significantly increased risk of cancer (RR 2.04; 95% CI, 1.11–3.74), although the authors argued that this result may simply reflect the small numbers for the age- and sex-stratified analyses rather than the real effect of T1DM on cancer incidence.^13^ The study by Shu et al^12^ also demonstrated that gender was a key factor in excess cancer incidence. After exclusion of the first year following T1DM diagnosis, SIR remained increased only among women. The study by Harding et al showed that the SIRs of all-cause cancer in males and females were 1.02 (95% CI, 0.96–1.09) and 1.10 (95% CI, 1.04–1.17), respectively.^28^ The SIRs for males were significantly increased for cancer of the pancreas, liver, and esophagus. Further, in contrast to our data, they found a reduced risk of prostate cancer in male T1DM patients.

It is very difficult to compare our results to the above findings of previous studies, mainly because of dissimilarities in T1DM case definition and length of follow-up. The poor prognosis and survival in male T1DM patients could also be responsible for the observed gender difference in SIR of cancer incidence noted in this study. It is well-recognized that both cardiovascular disease (CVD) and renal disease are major causes of death in patients with diabetes. The absence of nephropathy after 15–20 years of diabetes onset could be a marker of long-term survival of patients with diabetes.^29^ In a discrete-time simulation model using published information on mortality from CVD, renal disease, and risk of lower-extremity amputation and blindness to estimate and compare mean life expectancy and quality-adjusted life-years in patients treated for T1DM, researchers showed that from 15 years of age, male and female patients had respective estimated life expectancies of 47.2 (95% CI, 35.2–59.2) and 52.7 (95% CI, 41.7–63.6) years in the intensive treatment group.^30^ A higher risk of mortality from CVD and renal disease in male T1DM patients may compete with the risk of cancer incidence, leading to lower SIR in male T1DM patients than in females.

T2DM is associated with elevated risk of liver, pancreas, endometrium, colonrectum, breast, and bladder cancer, and with a decreased risk of prostate cancer.^1^ Site-specific cancer incidence in relation to T1DM varied in previous studies, and findings are inconclusive. A number of cancer types were found to be associated with T1DM, including pancreatic cancer,^13^^,^^28^^,^^31^^,^^32^ liver cancer,^14^^,^^28^ oropharyngeal cancer,^14^^,^^28^ gastric cancer,^12^ skin cancer,^12^ leukemia,^12^ ovarian cancer,^28^^,^^33^ endometrial cancer,^28^ colorectal cancer,^28^ thyroid cancer,^28^ brain cancer,^28^ and lung cancer.^28^ One study reported a decreased risk of prostate cancer and melanoma.^28^ Our study showed that T1DM is associated with significantly increased risks of colon and pancreatic cancer in both genders. In addition, T1DM was associated with more than two-fold increase in risks of prostate and kidney cancers in men, as well as bladder and ovarian cancers in women. It is not clear what may have accounted for the inconsistent findings on site-specific risk estimates for cancer incidence in the present study. However, the lack of information on potential confounders and the small number of observed incident cancers for each specific site are possible explanations.

The mechanism involved in the neoplastic process associated with T1DM remains unclear. The observed association between T1DM and cancer could be directly related to hyperglycemia or could be a marker of certain underlying biologic factors that alter cancer risk (eg, insulin resistance).^1^^–^^3^ Additionally, patients with T1DM are usually characterized by pancreas β-cell autoimmunity with insulin deficiency that normally requires lifelong provision of exogenous insulin, which is absorbed directly into systemic circulation. For sufficient levels of insulin in portal circulation to suppress hepatic gluconeogenesis, supraphysiologic concentrations of insulin should be reached in systemic circulation.^23^ The iatrogenic insulin excess and possible mutagenic effect of insulin or insulin analogue might account for some increased cancer risk.^1^^,^^34^^–^^36^

The role of genetic factors on the correlation between diabetes and cancer is not clear. One of the main risk variants associated with T2DM, *TCF7L2*, was also associated with colorectal cancer.^37^^,^^38^ Additionally, two genetic determinants of T2DM, *HNF1B* and *JAZF1*, were found to be associated with lower risk of prostate cancer.^39^ In contrast to T2DM, very few studies have reported the genetic correlation between T1DM and cancer. Takahashi et al identified 12 miRNAs from T1DM patients that were also present in cancer cell lines studied by Vaz et al.^40^^,^^41^ Such correlations might be associated with the elevated cancer risk in T1DM patients. However, in conflict with these miRNA findings, Chung et al observed marked different genetic expression between T1DM and pancreatic cancer patients.^42^ For example, the activity of B7-H4, a coregulatory molecule involved in immune response, was decreased in T1DM patients but increased in those with pancreatic adenocarcinoma.^42^ Increased B7-H4 expression was also reported in studies involving breast, ovarian, kidney, lung, prostate, and gastric cancer, as well as melanoma.^42^ Although some genetic correlations between T1DM and cancer have been reported, the relationship is not well-established and needs further investigation.

The biologic mechanism by which T1DM may cause colon cancer has not been fully elucidated. Exogenous hyperinsulinemia induced by insulin therapy is prevalent in T1DM patients and may be an important factor promoting growth of colonic epithelial cells^43^ via direct activation of insulin receptor or insulin-like growth factor (IGF)-I receptor and inhibition of IGF binding protein.^44^ Elevated levels of C-peptide, a marker of insulin secretion, have been found to be related to increased risk of colorectal cancer in many studies.^5^^,^^6^ Further, chronic insulin therapy was reported to be associated with an increased risk of colorectal adenoma and carcinoma.^45^

Similar to our present findings, many previous studies have found significant associations between T1DM and pancreatic cancer. An earlier meta-analysis showed a two-fold increase in risk of pancreatic cancer in patients with type 1 and young-onset diabetes.^31^ A recent meta-analysis included newer studies and also indicated a two-fold risk of pancreatic cancer in diabetic patients.^32^ The possible underlying biological mechanism by which T1DM may cause malignant neoplasm of the pancreas has not been fully elucidated, although insulin resistance, hyperglycemia, hyperinsulinemia, and inflammation have been suggested as potential mechanisms.^46^ Both T1DM and T2DM showed approximately two-fold increases in risk of pancreatic cancer, despite differences in pathophysiology between them.^31^^,^^47^ The core defects related to T2DM are insulin resistance and the compensatory hyperinsulinemia.^48^

In contrast to T2DM, T1DM might still present with insulin resistance but with negligible endogeneous insulin secretion.^49^ This phenomenon may imply that other factors, such as hyperglcyemia and inflammation, could also be alternative mechanisms for pancreatic carcinogensis related to T1DM. Alternatively, the correlation between diabetes and pancreatic cancer might be somewhat related to reverse causality, as the undiagnosed pancreatic cancer may induce insulin resistance and thereby cause diabetes (ie, type 3c diabetes). Green and Jensen^13^ found a significantly increased risk of pancreatic cancer, with a RR of 2.53, but the RR attenuated to 1.69 and became insignificant (*P* = 0.29) once cases where diabetes was an indicator of cancer were excluded, which provided some evidence that the observed association between T1DM and pancreatic cancer might be an artifact, at least to some extent, of reverse causality.

The present study has several strengths. First, we employed a nationwide population-based cohort design that included almost all patients with T1DM during the study period, which leaves little room for selection bias. Second, many of the previous studies used different criteria to identify T1DM, such as age at first diagnosis or age at first hospitalization for diabetes, and age used to determine T1DM ranged from disease diagnosis from ages <21^12^ to those <40 years old.^16^ Although it is likely that the younger classification of T1DM is more likely to capture a cohort which most closely matches the true T1DM population, disease misclassification may still exist, given the increasing epidemic of T2DM in the youth. For example, a Japanese study surveyed schoolchildren and found that T2DM is seven times more common than T1DM.^50^ However, unlike previous studies that used an arbitrarily selected age to determine T1DM, our study used the medical claim with CICs to identify patients with T1DM, which ensures that the information of T1DM in this study was valid.^18^

Despite the above strengths, certain limitations are involved in our study and should be mentioned. First, although we managed to adjust for age, gender, and calendar year in calculating the relative risk estimates of cancer in relation to T1DM, no information concerning the potential risk factors for cancer, such as physical inactivity, family cancer history, smoking, and alcohol consumption, is available from Taiwan’s NHI claim data. Second, one previous study found that some 23% of patients with T1DM in Taiwan were admitted during the first year of T1DM diagnosis between 2003 and 2007.^51^ On the other hand, only 8% of the general population were admitted in 2005.^52^ This information suggests that screening or surveillance bias might be a concern in our study, because there are more frequent physician contacts for patients with T1DM. However, there is some evidence that surveillance bias may have little effect on the estimates. One earlier Taiwanese study noted an increased risk of liver cancer in patients with T2DM. In order to assess whether the significant association of T2DM with malignant neoplasm of the liver was due to frequent surveillance of disease among diabetic patients, the authors limited the control subjects to hypertensive subjects who can also be considered frequent clinic visitors. The results showed that the recalculated point estimates of hazard ratios (HRs) for malignant neoplasm of the liver (HR 1.28) and biliary tract (HR 1.02) were very similar to the original estimates (HR 1.21 and 1.07, respectively).^53^ Similar findings have also been observed in studies of breast cancer^54^ and ovarian cancer^55^ in patients with T2DM, suggesting little surveillance bias in Taiwan’s clinical setting.

### Conclusion

T1DM was associated with a 13% increase in the risk of all-cause cancer incidence. Patients with T1DM should be advised to undergo cancer screening for certain types of cancer.
